# Secular trends in childhood pain and comorbid psychiatric symptoms: a population-based study

**DOI:** 10.1007/s00127-022-02234-w

**Published:** 2022-02-18

**Authors:** Terhi Luntamo, Lotta Lempinen, Andre Sourander

**Affiliations:** 1 Department of Child Psychiatry, University of Turku and Turku University Hospital, Turku, Finland; 2 INVEST Research Flagship Center, University of Turku, Turku, Finland

**Keywords:** Child, Epidemiology, Psychiatry, Pain

## Abstract

**Purpose:**

Pain symptoms are common in childhood. They often lead to functional impairment and co-occur with psychiatric difficulties. Although children’s lives have undergone enormous changes in recent decades, long-term data on changes in pain symptoms, and in comorbid psychiatric difficulties, is lacking. This knowledge is crucial, as co-occurring psychiatric symptoms are significant predictors of long-term outcome for children who suffer from pain. The main purpose of the present study was to explore secular changes in comorbid pain and psychiatric symptoms.

**Methods:**

Four population-based, cross-sectional surveys of 8–9-year-old children were conducted in Southwest Finland in 1989, 1999, 2005, and 2013. Identical methodologies and questionnaire-based measures were used each study year. Participation ranged from 891 to 986 over the study period. The children were asked about the frequency of headache, abdominal pain, and other pains. Children, their parents, and teachers provided information on the child’s psychiatric difficulties, including internalizing and externalizing symptoms.

**Results:**

The cumulative odds ratios and 95% confidence intervals for the overall prevalence of pain symptoms increased among both genders from 1989 to 2013 and ranged from 1.4 (1.03–1.8) for other pains to 2.4 (1.7–3.3) for abdominal pain. Comorbid internalizing symptoms increased among girls with odd ratios and 95% CIs of 1.8 (1.03–3.1) for children with any kind of pain, and 3.0 (1.4–6.2) for children with headache. No changes were found among boys.

**Conclusion:**

Overall pain symptoms doubled in both genders, but the most novel finding was that comorbid emotional difficulties tripled among girls who reported headaches. Further research is needed to confirm, and explain, these findings.

## Introduction

Pain is defined as an unpleasant sensory and emotional experience associated with, or resembling that associated with, actual or potential tissue damage. It is always a personal experience and influenced by biological, psychological, and social factors [1].

Different kinds of pains are among the most common health complaints in childhood [2]. Among school-aged children, headache is the most commonly reported pain symptom, followed by abdominal pain and musculoskeletal pain, such as back pain and limb pain. In large-scale population-based studies conducted in Western countries, the prevalence of recurrent headache, abdominal pain, and back pain have varied from 10 to 48%, from 3 to 39%, and from 1 to 25%, respectively [2–6]. Childhood pain is a common reason for healthcare visits [7], and considerably decreases children’s quality of life. It may impair cognitive functions, distract children for example from meeting friends and pursuing hobbies, and lead to school absenteeism [8–11]. It additionally predicts later pain as well as psychiatric problems up until adulthood [12, 13].

Psychological factors are considered to play a significant role in the onset, and particularly continuity and impact of pain. The experience of pain has been associated with psychosocial stress and negative life events, as well as with dysfunctional cognitive styles, such as catastrophizing and anxiety sensitivity [10, 14–17]. A cognitive model [18] proposes that individuals who suffer from recurrent pain are overly worried about it and its consequences. This leads to emotional arousal and stress, as well as an increased amount of attention directed to the symptom and the factors that might make it worse. These processes, together, lead to over-evaluation of the problem and to increased symptom levels, thus creating a vicious cycle. Recurrent pain has also been associated with children’s psychiatric difficulties and disorders in clinical and population-based samples. This concerns particularly emotional problems, including anxiety and depression [14, 19–22]. Pain may lead to psychiatric difficulties, mediated for example by the daily difficulties it leads to at home, school, and with friends [23]. The other way around, pain is commonly known as a symptom in a number of childhood emotional disorders. Additionally, pain and emotional problems share several etiological factors, including the above-mentioned psychosocial stress, negative life events, and dysfunctional cognitive styles [10, 14–17, 24]. Comorbid psychiatric difficulties are highly relevant, as they predict poorer prognosis regarding the continuity of pain, pain-related disability, and quality of life [21, 25–29], even after controlling for the severity of the pain and number of somatic symptoms at baseline [26].

During the last few decades, several factors that have been associated with children’s well-being, and the occurrence of both pain and psychiatric symptoms, have undergone remarkable changes. These include family and school environments, leisure activities, and nutritional habits [30, 31]. There has also been speculation about how increased health monitoring, including seeking information on the Internet, may have affected people’s anxiety about health and illnesses. Health anxiety has increased among adults and students, which has potentially led to the increased use of health care services [32]. So far, knowledge about the time trends on emotional issues among children who have pain, is lacking.

To obtain reliable data, it is crucial to collect information repeatedly, using comparable study designs and methods. The World Health Organization has highlighted several studies about changes in adolescents’ pain symptoms [33]. However, these results cannot be applied to younger children, as they are not experiencing the same hormonal and lifestyle changes, and pressure of social role expectations, which have been reported to play a role among adolescents [9, 19]. Studies on secular trends in childhood pain in the general population are rare. Krause et al. [34] reported increased headaches, abdominal, and back pain among children aged 7–10 years, from 2003–2006 to 2014–2017, but the measures used were not identical at the two time points. Similarly, Santalahti et al. [35] showed a rise in headaches and abdominal pain among 8-year-old children from 1989 to 1999. However, changes between two time points could simply reflect a fluctuation over time. Only two studies were identified that used three comparable data collections. Anttila et al. [36] found that the risk of frequent headaches had more than doubled among 7-year-olds from 1974 to 2002. Similarly, a previous paper by our team reported that the prevalence of various pain symptoms almost doubled among 8–9-year-old children from 1989 to 2005 [22]. To our knowledge, no studies have addressed changes in the comorbidity of pain and psychiatric symptoms. Repeating studies on trends in children’s well-being can help to raise awareness of possible changes in health care service needs. Increasing trends would be meaningful, given the disadvantages that pain has for the child, the family, and society. Such changes would also highlight the need for studies addressing the underlying reasons for increasing symptom rates.

This study reports the results of a unique time-trend study, including four identical population-based cross-sectional surveys of 8–9-year-old children, and their teachers and parents, conducted in 1989, 1999, 2005, and 2013. It is based on large-scale epidemiological data that were originally collected to investigate young children’s psychiatric symptoms, and how they have changed over time [37]. Our aim is to report secular trends in the prevalence of pain symptoms and co-occurring psychiatric difficulties in childhood.

## Methods

### Study design, subjects, and procedure

**Fig. 1:**
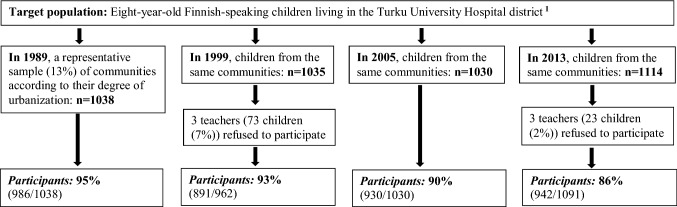
Study design, subjects, and response rates.* Note* ^1^Children who had severe intellectual disability were excluded from the study

The same method was used to gather four cross-sectional population-based samples, in 1989, 1999, 2005, and 2013. The study design is presented in Fig. 1.

The target population was 8–9-year-old Finnish-speaking pupils. They lived in the Turku University Hospital District in Southwest Finland, which covers about a fifth of the Finnish population. Most were in their second grade of primary school. The data in 1989 were gathered as a subsample of the national Epidemiologic Multicenter Child Psychiatric Study in Finland, which provided information on altogether 6017 children across the country [38]. It was carried out by all five Finnish universities that have medical faculties. At that time, data on young children’s mental health problems, as reported by the children, their parents and teachers, were largely lacking, and 8-year-old children were considered to be old enough to answer the questions. A representative sample of school districts in the area according to their degree of urbanization (urban, suburban, rural) was made in 1989, representing 13% of the target population. The study was planned to be as inclusive and representative as possible. Children attending special classes due to, for example, behavioral disturbances, physical handicaps, and mild intellectual disability, were included in the study. Only children who had severe intellectual disability were excluded. The study sample of 1989 was controlled for demographic and socioeconomic factors in the general population and was found to have good generalizability [38]. In 1999, 2005, and 2013, children from the same municipalities and school districts served as study samples. Similar numbers took part each year: 986 in 1989, 891 in 1999, 930 in 2005, and 942 in 2013. The breakdown by sex was fairly even.

The ethics committee of Turku University Hospital approved the study design before any data were collected. After that, authorities in charge of the school system in each county, as well as the principals and teachers, were informed about the study, its objectives and implementation. The data were gathered in November in 1989, 1999, and 2005, and in March in 2013. Each year, data collection comprised questionnaires completed by the child and their parents, as well as teachers. All three questionnaires were delivered to the teachers by the study group. The teachers distributed the questionnaires, together with the consent forms, to the parents via the children. Participation in the study was voluntary and the parents returned the questionnaires in sealed envelopes. In 1989, the completed questionnaire was considered as an informed consent, but due to legislative changes, formal, signed consent was required in the other three study years. The children and the teachers filled in their questionnaires during a class, so that the children could get help if they had difficulties to understand the questions. The children gave their completed questionnaires to the teachers in sealed envelopes. The teachers returned all three questionnaires to the researchers.

### Measures

The same measures were used each study year.

#### Pain symptoms

The questions on pain were specially designed for the study, because no validated instruments were available when the first data were collected in 1989. The children were asked how frequently they had headaches, abdominal pain and other pains during the past 2 weeks. The possible responses for each of these three questions were seldom, often or almost every day. Additionally, for the statistical analyses, we combined the often and almost every day responses to create an additional ‘any pain’ category, meaning that the child had either headache, abdominal pain or other kind of pain at least often.

#### Psychiatric symptoms

The children, parents, and teachers were asked about the child’s psychiatric symptoms. We evaluated both internalizing symptoms (anxiety, depression) and externalizing symptoms (conduct problems, hyperactivity). Internalizing symptoms were measured by asking the children to complete the Child Depression Inventory (CDI) [39]. This well-known measure has been developed to assess children’s depressive symptoms during the last 2 weeks and uses a simple three-point scale to measure 27 items from 0 to 2, with a maximum score of 54. The CDI measures emotional distress or disturbance well, but it is not as effective when it comes to distinguishing between depression and anxiety [40]. The item on suicidality was omitted, because we felt it would not be appropriate in a classroom setting. The CDI item about pains was also excluded before statistical analyses to avoid over-evaluation of the comorbidity between pain and emotional difficulties. This meant that the CDI used in this study comprised 25 items, and the score ranged from 0 to 50, with higher scores indicating more symptoms. Externalizing symptoms, namely conduct problems and hyperactivity, were assessed using the Rutter Parent and Teacher Questionnaires [41, 42]. These are widely used, valid screening methods that assess a child’s psychiatric difficulties over the past 12 months. Conduct problems were covered by five items for parents and six items for teachers, and included symptoms such as disobedience, lying, stealing, and aggressive behavior. Both questionnaires include the same three items on hyperactivity, which are restlessness, inattention, and irritability. Each item was scored from 0 to 2, with higher scores indicating more symptoms. In line with earlier studies [43], we used the sex-specific 90th percentile cut-off scores for the internalizing symptoms, as well as conduct problem and hyperactivity symptom subscales. The psychiatric symptoms were divided into two categories for the analyses: 1. The child was considered to have internalizing symptoms when scoring over the 90th percentile, based on the self-report. 2. The child was considered to have externalizing symptoms when scoring over the 90th percentile in either conduct or hyperactivity symptoms, according to at least one reporter, parent and/or teacher. When comparing parent and teacher reported externalizing symptoms, the agreement was *α* = 0.37 for boys and α = 0.25 for girls, which means fair agreement. Among boys, teachers reported more symptoms (mean: 3.0 points) than parents (mean: 2.1 points). Among girls, parents reported slightly more symptoms (mean: 1.3 points) than teachers (mean: 0.9 points).

#### Demographics

The demographic information that were included in the analyses as covariates were the family structure and the mother’s vocational educational level. Family structure was divided into: (1) living with two biological parents or (2) any other type of family composition. Maternal vocational educational level was divided into: (1) a college or university degree or (2) a lower level of education. The information on who filled in the parental questionnaire was reliably available only in 2005 and in 2013. In 2005, 79% of the respondents were mothers, and 10% were fathers. In 2013, the mother filled in the questionnaire for 67%, the father for 6%, and the parents together for 20% of the children. For the rest of the children, the information was missing, or the questionnaire was filled in by someone else, such as a grandparent.

#### Statistical methods

Changes in the prevalence of pain symptoms between the study years were studied with cumulative regression analysis if there were three categorical response variables, and with logistic regression if there were two. Interactions between year and gender were studied for different symptoms. The analyses were adjusted with gender, family composition and the mother’s education. Differences in pain symptoms between genders were studied with logistic regression. Odds ratios (ORs), cumulative ORs (CORs), and 95% confidence intervals (95% CIs) were calculated. Subgroups of children who reported headaches, abdominal pains, other pains, or any kind of pain, were created, and changes in internalizing and externalizing symptoms between years in these pain groups were studied with logistic regression. The agreement between parent and teacher reported externalizing symptoms was studied with McNemar’s test and Kappa coefficient. The level of statistical significance was a two-sided *p* value of < 0.05 in most of the analysis. The exception was the interaction analyses, where *p* < 0.1 was used. All the analyses were carried out using SAS for Windows, version 9.4 (SAS Institute, Cary, NC, USA).

## Results

### Prevalence changes of pain symptoms

**Fig. 2:**
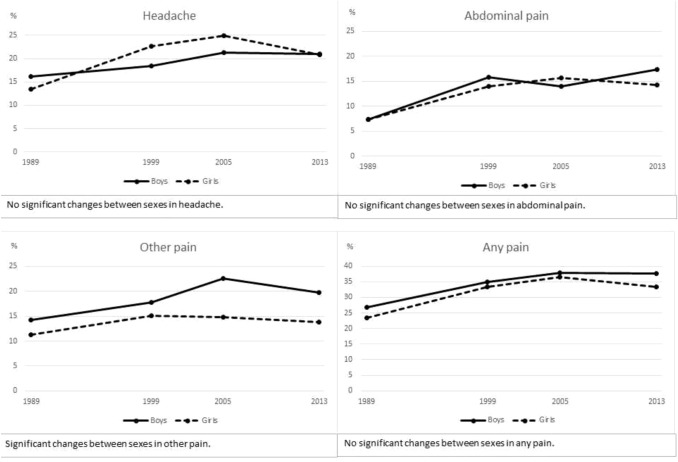
Prevalence of any pain^1^—headache, abdominal pain or other pain—in 1989, 1999, 2005, and 2013 among boys and girls.* Note*: ^1^Any pain refers to having pain symptom often or almost every day

Changes in the prevalence of pain symptoms from 1989 to 2013 are presented in Fig. 2 and Table 1. Figure 2 shows the prevalence for boys and girls in each study year. There were no significant differences in the prevalence of headaches or abdominal pains between the genders, but boys had more other pains than girls. In addition, there were no significant interactions between gender and study year for pain symptoms. That is why the results are reported jointly for boys and girls.

**Table 1:** Changes in the prevalence of pain symptoms from 1989 to 2013

	1989 (*n* = 912)	1999 (*n* = 628)	2005 (*n* = 784)	2013 (*n* = 891)	OR/COR (95% CI)^a^ 1999 vs 1989	OR/COR (95% CI)^a^ 2005 vs 1989	OR/COR (95% CI)^a^ 2013 vs 1989
Any pain* (%)	25.1	34.1	37.2	35.7	**1.5 (1.2–1.9)**^b^	**1.8 (1.4–2.3)**^b^	**1.6 (1.3–2.0)**^b^
Headache							
Often (%)	11.9	14.1	17.9	15.5			
Almost daily (%)	2.8	6.5	5.3	5.5	**1.4 (1.1–1.9)**^c^	**1.8 (1.4–2.4)**^c^	**1.6 (1.2–2.1)**^c^
Abdominal pain							
Often (%)	5.1	12.5	11.8	11.2			
Almost daily (%)	2.2	2.4	3.0	4.8	**2.0 (1.4–2.9)**^c^	**2.2 (1.6–3.1)**^c^	**2.4 (1.7–3.3)**^c^
Other pains							
Often (%)	10.2	12.6	14.8	13.8			
Almost daily (%)	2.6	3.8	3.8	3.3	1.3 (0.9–1.7)^c^	**1.7 (1.2–2.2)**^c^	**1.4 (1.03–1.8)**^c^

The numbers refer to the number of children who responded to the pain questions in each study year

Statistically significant results are in bold (*p* value < 0.05)

*Any pain: children reported having headache, abdominal pain or other pains often or almost every day

^a^Adjusted for gender, family structure, and maternal education

^b^OR, odds ratios

^c^COR, cumulative odds ratios; 95% CI, 95% confidence interval

Pain symptoms increased significantly from 1989 to 2013 (Table 1). About a fourth of the children had pain often or almost every day in 1989 (25.1%), and this had increased to about a third in 2013 (35.7%). The CORs and 95% CIs for the increase in the symptom levels varied from 1.4 (1.03–1.8) for other pains to 2.4 (1.7–3.3) for abdominal pain. As the results remained similar after adjustment with family structure and maternal education level, only the final models are presented. There were no significant changes in the prevalence of pain between the last two study years, 2005 and 2013.

### Prevalence of comorbid psychiatric symptoms and childhood pain 

Table 2 presents the changes in internalizing and externalizing symptoms from 1989 to 2013 among children who reported pain. As there were significant interactions between gender and year, the results are reported separately for boys and girls.

**Table 2:** Prevalence changes of internalizing and externalizing symptoms among boys and girls who experienced pain, from 1989 to 2013

	Boys				Girls			
	1989	1999	2005	2013	1989	1999	2005	2013
Any pain*								
Internalizing (%)	27.1	24.5	16.9	26.3	24.3	27.6	37.8	36.5
OR (95% CI)^a^	1	0.9 (0.5–1.6)	**0.5 (0.3–0.99)**	0.96 (0.6–1.6)	1	1.2 (0.6–2.2)	**1.9 (1.1–3.3)**	**1.8 (1.03–3.1)**
OR (95% CI)^b^	1	1.0 (0.5–2.0)	0.7 (0.4–1.4)	1.1 (0.6–2.0)	1	1.1 (0.6–2.1)	**1.8 (1.04–3.3)**	**1.8 (1.02–3.2)**
Externalizing (%)	41.0	34.0	32.0	38.7	37.3	35.3	46.2	48.1
OR (95% CI)^a^	1	0.7 (0.4–1.3)	0.7 (0.4–1.1)	0.9 (0.6–1.5)	1	0.9 (0.5–1.6)	1.4 (0.9–2.4)	1.6 (0.9–2.6)
OR (95% CI)^b^	1	0.8 (0.4–1.4)	0.7 (0.4–1.3)	0.9 (0.5–1.5)	1	0.9 (0.5–1.7)	1.5 (0.9–2.6)	1.6 (0.9–2.7)
Headache								
Internalizing (%)	33.8	26.8	18.8	32.7	20.3	25.7	37.3	43.0
OR (95% CI)^a^	1	0.7 (0.3–1.5)	**0.5 (0.2–0.95)**	0.95 (0.5–1.8)	1	1.4 (0.6–3.1)	**2.3 (1.1–4.8)**	**3.0 (1.4–6.2)**
OR (95% CI)^b^	1	0.8 (0.3–1.8)	0.5 (0.2–1.2)	0.95 (0.5–2.0)	1	1.2 (0.5–2.9)	**2.2 (1.03–4.9)**	**3.0 (1.4–6.5)**
Externalizing (%)	45.7	42.0	33.8	37.2	36.5	32.4	47.8	53.0
OR (95% CI)^a^	1	0.9 (0.4–1.8)	0.6 (0.3–1.2)	0.7 (0.4–1.3)	1	0.8 (0.4–1.7)	1.6 (0.8–3.1)	**2.0 (1.004–3.8)**
OR (95% CI)^b^	1	0.8 (0.4–1.7)	0.6 (0.3–1.3)	0.7 (0.3–1.4)	1	0.8 (0.4–1.8)	1.7 (0.8–3.4)	2.0 (0.97–4.0)
Abdominal pain								
Internalizing (%)	25.0	39.6	24.5	34.5	34.3	34.8	50.0	39.0
OR (95% CI)^a^	1	2.0 (0.7–5.3)	0.98 (0.4–2.7)	1.6 (0.6–4.0)	1	1.0 (0.4–2.6)	1.9 (0.8–4.5)	1.2 (0.5–2.9)
OR (95% CI)^b^	1	**3.3 (1.02–10.9)**	1.8 (0.5–5.7)	2.4 (0.8–7.3)	1	1.1 (0.4–2.9)	1.7 (0.7–4.3)	1.4 (0.5–3.4)
Externalizing (%)	43.8	41.9	33.3	42.0	40.0	34.9	51.7	55.2
OR (95% CI)^a^	1	0.9 (0.4–2.3)	0.6 (0.3–1.6)	0.9 (0.4–2.1)	1	0.8 (0.3–2.0)	1.6 (0.7–3.7)	1.8 (0.8–4.3)
OR (95% CI)^b^	1	1.0 (0.4–2.9)	0.8 (0.3–2.2)	1.1 (0.4–2.7)	1	0.8 (0.3–2.2)	1.5 (0.6–3.7)	1.9 (0.8–4.7)
Other pains								
Internalizing (%)	28.6	27.8	16.5	33.7	33.3	39.6	40.0	49.1
OR (95% CI)^a^	1	0.96 (0.4–2.2)	0.5 (0.2–1.1)	1.3 (0.6–2.5)	1	1.3 (0.6–2.9)	1.3 (0.6–2.9)	1.9 (0.9–4.2)
OR (95% CI)^b^	1	1.3 (0.5–3.2)	0.6 (0.3–1.5)	1.5 (0.7–3.2)	1	1.1 (0.5–2.7)	1.4 (0.6–3.1)	2.3 (0.999–5.2)
Externalizing (%)	36.5	34.0	31.2	47.2	47.2	43.5	50.9	57.1
OR (95% CI)^a^	1	0.9 (0.4–2.0)	0.8 (0.4–1.6)	1.6 (0.8–3.0)	1	0.9 (0.4–1.9)	1.2 (0.5–2.5)	1.5 (0.7–3.2)
OR (95% CI)^b^	1	0.9 (0.4–2.1)	0.8 (0.4–1.8)	1.5 (0.7–3.0)	1	0.8 (0.4–2.0)	1.2 (0.5–2.7)	1.5 (0.7–3.4)

Statistically significant results are in bold (*p* value < 0.05)

Internalizing symptoms: scores above the 90th percentile in the CDI, reported by the child. Externalizing symptoms: scores above the 90th percentile in the conduct or hyperactivity subscale of the Rutter scale, reported by the parent and/or teacher. The reference group was children with pain, but no psychiatric symptoms

OR, odds ratios; 95% CI, 95% confidence interval

*Any pain: children reported having headache, abdominal pain or other pain often or almost every day

^a^Unadjusted results

^b^Adjusted for family structure and maternal education

No significant changes were found in the prevalence of comorbid internalizing or externalizing symptoms from 1989 to 2013 among boys who had pain. However, there was an increase in internalizing symptoms among girls who reported pain, particularly headaches. The OR in 2013 was 3.0 (95% CI 1.4–6.2) compared to 1989 among girls with headache, and 1.8 (95% C I 1.03–3.1) among girls with any kind of pain. In addition, there was a significant increase in externalizing symptoms among girls who had headaches (OR 2.0, 95% CI 1.004–3.8). No changes were found in internalizing or externalizing symptoms among girls who suffered from abdominal or other pains.

When the results were adjusted for the demographic variables of family structure and maternal education level, the increase in internalizing symptoms among girls with any pain, and particularly headaches remained practically unchanged. The increase in externalizing symptoms among girls with headaches was no longer statistically significant.

## Discussion

This Finnish study compared the prevalence of pain symptoms and comorbid psychiatric difficulties in 1989, 1999, 2005, and 2013, by collecting data from 8–9-year-old children, and their teachers and parents. Identical measures and study procedures were used. There were three main findings. First, the prevalence of pain symptoms increased among both genders from 1989 to 2013, but remained stable in the last two study years, 2005 and 2013. Second, there were no changes in the prevalence of comorbid psychiatric symptoms among boys. Third, the amount of comorbid internalizing symptoms among girls who reported pain, and particularly headaches, showed a remarkable increase from 1989 to 2013.

Earlier time-trend studies on the prevalence of childhood pain reported increasing trends [22, 35, 36]. However, only one study was carried out until the 2010s. Krause et al. [34] found an increase in the prevalence of headache, abdominal pain and back pain among children aged 7–10 years, when they compared data from the German Health Interview and Examination Survey for Children and Adolescents in 2003–2006 and 2014–2017. The authors evaluated recurrent pain using parental reports, whereas we used child self-reports. In addition, the two German surveys differed slightly when it came to the questions they asked about pain. The differing results between that study and ours may be due to methodological differences, or different recent trends in Germany and Finland.

To our knowledge, this study is the first to report data on changes in comorbid psychiatric difficulties among children who have pain. It is interesting that no changes were seen among boys, whereas girls reported increased comorbid psychiatric symptoms from 1989 to 2013. A remarkable finding was that comorbid internalizing difficulties almost doubled among girls who had any kind of pain, and even tripled among girls who had recurring headaches. It is possible that headache is more likely to be a marker for psychological stress, or that it causes greater anxiety than other types of pain. This finding is in line with our earlier study showing that headaches predicted later antidepressant use, but abdominal pain and other pains did not [13]. One possible explanation for the highest rate of comorbid emotional difficulties among girls with headache could also be that headaches and internalizing symptoms share a common genetic vulnerability to some degree [44].

The present study does not provide reasons for the increased comorbidity of pain and emotional difficulties among girls. Pain has been reported to be more common among adolescent girls than boys [14], but this does not apply to younger children [22]. Additionally, although the prevalence of emotional difficulties has increased among adolescent girls over recent decades [45], no increase in emotional or behavioral symptoms have been reported for 8-year-old Finnish children [37]. Thus, the present findings cannot be due to an increased prevalence of psychiatric symptoms.

However, girls are more likely to associate pain with emotional distress, whereas boys are more likely to blame physical problems [8]. In addition, the association between pain and functional disability seems to be stronger among girls [8, 46, 47]. Functional disability may impair for example friendships and academic performance, which may, in turn, increase psychiatric difficulties. There were changes in Finnish preventive health care, school system, and school support services during our study period [30]. These led to issues such as increased number of children in classes and higher academic pressure, as well as decreased possibilities for early recognition and interventions. In addition, increased social pressure, including the use of social media, could play a role, particularly among girls [48]. The combination of these factors may have increased the burden of pain and/or psychiatric difficulties, thus leading to additional symptoms. Furthermore, more digital information about somatic diseases could have increased anxiety [32]. Pain catastrophizing is more common among girls, as boys are more likely to use positive strategies, such as behavioral distraction [49]. Pain catastrophizing, in turn, has been associated with general anxiety and depression [10]. These differences in the interpretation and impact of pain among boys and girls might explain the findings.

### Strengths and limitations

Strengths of the study include measuring the same symptoms four times during a 24-year time period, and the use of identical study procedures and measures to produce comparable data. We asked children, and their parents and teachers about the child’s psychiatric symptoms. Using several informants is generally recommended in child psychiatry, as children can display diverse symptoms in different surroundings. In addition, children are more likely to be aware of internalizing symptoms, and parents and teachers are more likely to notice externalizing symptoms. The responses were high and the characteristics of the study subjects were representative of the target population.

However, there were some limitations. The data collections in 1989, 1999, and 2005 took place in November, whereas the last data collection in 2013 was due to practical reasons later, in March. Thus, it is possible that this difference may have induced the steadiness that was found in the symptom levels between 2005 and 2013. Previous studies have reported seasonality, possibly due to differences in light exposure, climate, and circadian regulation, for both childhood pain as well as psychiatric problems [50–52]. We only asked the children about the frequency of pain in the last 2 weeks and did not explore the intensity or duration of the pain, or any functional impairment. This was because no validated pain instruments were available in 1989, when the first study was carried out, and as the measures were to be kept identical when studying the time trends. Therefore, it is possible that changes in these pain domains could have explained the increased comorbidity with psychiatric symptoms. As the study was conducted in a classroom situation, the questionnaires were to be kept as simple and short as possible. Therefore, the pain items were formulated in a similar way as the items in the CDI are. The most common childhood pain symptoms, i.e., headache and abdominal pain, were included as distinct items, but unfortunately, it was not possible to make any distinctions between different kinds of “other pains”, such as back pain or limb pain. Additionally, pain items did not include an answer option “not at all”. Thus, in the present study, answer option “seldom” was considered as having no pain, and only frequent or almost daily pain was considered as having pain symptoms. In case of any uncertainty, the children had a possibility to ask their teacher about the meaning of the items and answer options. However, it is still possible that some children who reported for example headache, might have had pain in a more specific location, such as ear or tooth, which would not have much to do with psychiatric problems. However, as the items were identical each study year, there seems to be no reason to assume that this would have significantly affected the findings in regards to changes in time.

## Conclusion

The prevalence of childhood pain symptoms doubled from 1980 to 2010s, and the association between pain and comorbid internalizing symptoms remarkably strengthened among girls. These findings are of importance and call for research to address possible reasons for the increase, and prevention of childhood pain.

## Data Availability

The data that support the findings of this study are available from the corresponding author upon reasonable request.

## Abbreviations

95% CI: 95% Confidence interval
CDI: Child Depression Inventory
COR: Cumulative odds ratio
OR: Odds ratio
